# Two-stage surgical treatment of Stanford type B acute aortic dissection associated with aberrant right subclavian artery dissection complicated by distal arch aortic aneurysm and abdominal aortic aneurysm: a case report

**DOI:** 10.1186/s44215-024-00137-7

**Published:** 2024-02-25

**Authors:** Yusuke Shintani, Satoru Tobinaga, Hiroyuki Saisho, Takanori Kono, Eiki Tayama, Shigeaki Aoyagi, Hiroshi Yasunaga

**Affiliations:** 1 Department of Cardiovascular Surgery, St. Mary’s Hospital, Tsubuku Hon-machi 422, Kurume, 830-8543 Japan; 2 Department of Surgery, Kurume University, Asahi-machi 67, Kurume, 830-0011 Japan

**Keywords:** Aberrant right subclavian artery, Type B aortic dissection, Two-staged open surgery

## Abstract

**Background:**

An aberrant right subclavian artery (ARSA) is the most common congenital variant of the aortic arch and occurs in 0.5–1.8% of the population. Most patients with ARSA remain asymptomatic; however, symptoms associated with ARSA include dysphagia, esophageal compression, and airway obstruction. Surgical intervention is indicated if the ARSA becomes symptomatic or is related to aneurysmal dilatation. Even without symptoms, it carries the risk of rupture or dissection, and aggressive surgical therapy is recommended. The coexistence of type B dissection and ARSA is relatively rare, and the cause of this anomaly is unclear; however, some authors have reported that the acute angle of the ARSA weakens the aortic wall, inducing aortic dissection. Several surgical methods, such as thoracic endovascular aortic repair (TEVAR), the frozen elephant trunk method, and open surgery, have been used to manage this lesion. Reconstruction of ARSA is challenging in any surgical procedure.

**Case presentation:**

We present an uncommon case of coexistent type B aortic dissection and aberrant right subclavian artery (ARSA) in a 72-year-old man. Left anterolateral thoracotomy was chosen to treat the enlarged descending thoracic aortic aneurysm in this case; in situ reconstruction was difficult because the dissection involved the ARSA. Hence, preoperatively, a right common carotid artery (RCCA)-to-ARSA bypass was performed via the right supraclavicular approach, followed by thoracic descending aortic artery reconstruction. The prior RCCA-to-ARSA bypass allowed ligation of the central side of the ARSA, thereby securing a bloodless field in the distal anastomosis.

**Conclusion:**

This lesion can be successfully repaired by open surgery with a two-stage approach: right common carotid artery-to-ARSA bypass followed by thoracic aortic replacement.

## Background

An aberrant right subclavian artery (ARSA) is a common congenital anomaly of the aortic arch occurring in 0.5–1.8% of the population [1]. Most patients with an ARSA remain asymptomatic; however, an aberrant subclavian artery with Kommerell diverticulum (KD) is associated with the risk of rupture or dissection [2, 3].

Coexistent type B dissection with ARSA is relatively rare [1]. Various surgical techniques, such as thoracic endovascular aortic repair, hybrid approach, and open surgery, have been used to treat this complex aortic lesion [1]. Additionally, with rapid advancements in endovascular technology, total endovascular or hybrid procedures are being increasingly applied; nonetheless, a standard surgical procedure for this complex lesion has not yet been established.

Herein, we present a case of coexisting type B dissection and ARSA managed with open surgery using a two-stage approach (right common carotid artery [RCCA]-to-ARSA bypass followed by thoracic aortic replacement) in an adult patient.

## Case presentation

A 72-year-old man, with a history of hypertension but no history of dysphagia or dyspnea, was referred to our hospital with a sudden onset of back pain.

**Fig. 1:**
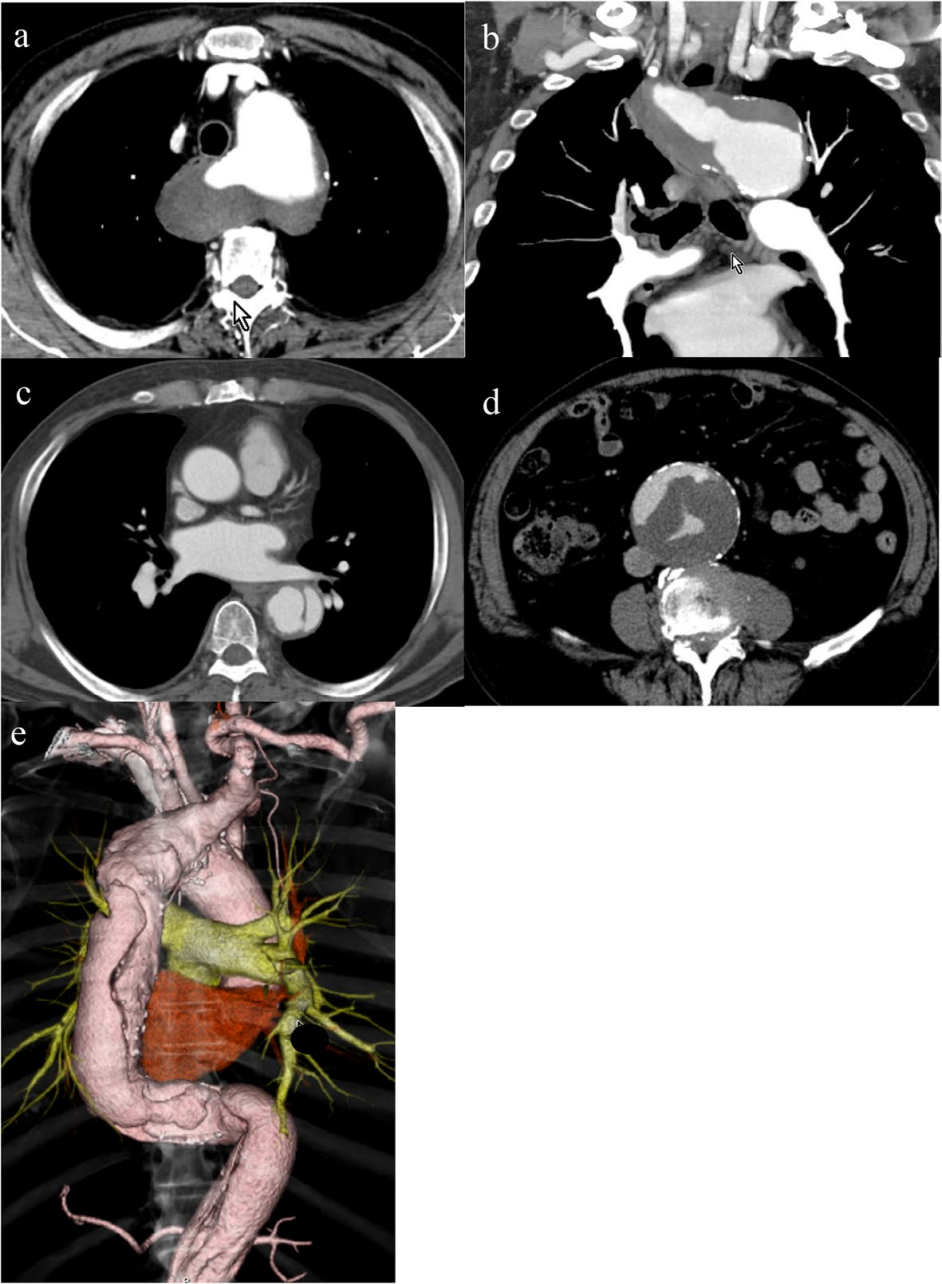
Enhanced computed tomography findings. **a**, **b** The aberrant right subclavian artery branched from the descending thoracic artery and its origin was dissected and dilated. **c** Aortic dissection of partial thrombosis type in the descending thoracic aorta. **d** Aortic dissection involving an abdominal aortic aneurysm. **e** 3D computed tomography reconstruction shows the aberrant right subclavian artery running distal to the left subclavian artery

On admission, his blood pressure was 144/92 mmHg and symmetrical in the upper extremities. Electrocardiography revealed no ischemic changes. Echocardiography revealed no valve abnormality or pericardial effusion. The ascending aorta was slightly dilated, but an intimal flap was not detected. Computed tomography (CT) revealed a left-sided aortic arch associated with an ARSA passing posterior to the esophagus; type B aortic dissection with partial thrombosis in the false lumen extending from the proximal thoracic descending aorta distal to the origin of the left subclavian artery (LSA) to the thoracoabdominal aorta immediately proximal to the renal arteries, including the origin of the ARSA; and a distal aortic arch aneurysm (56 mm). The ARSA arose from the posteromedial aspect of the proximal descending aorta as the fourth branch of the aortic arch, and its enlarged origin (47 mm) compressed the esophagus (Fig. 1). An aorto-iliac aneurysm (72 mm) was also detected (Fig. 1).

A diagnosis of acute type B dissection, distal aortic arch aneurysm, KD with the ARSA, and aorto-iliac aneurysm was made. After anti-hypertensive therapy, the aorto-iliac aneurysm was initially replaced with a bifurcated vascular graft. No extension of the dissection into the aneurysm was observed intraoperatively.

**Fig. 2:**
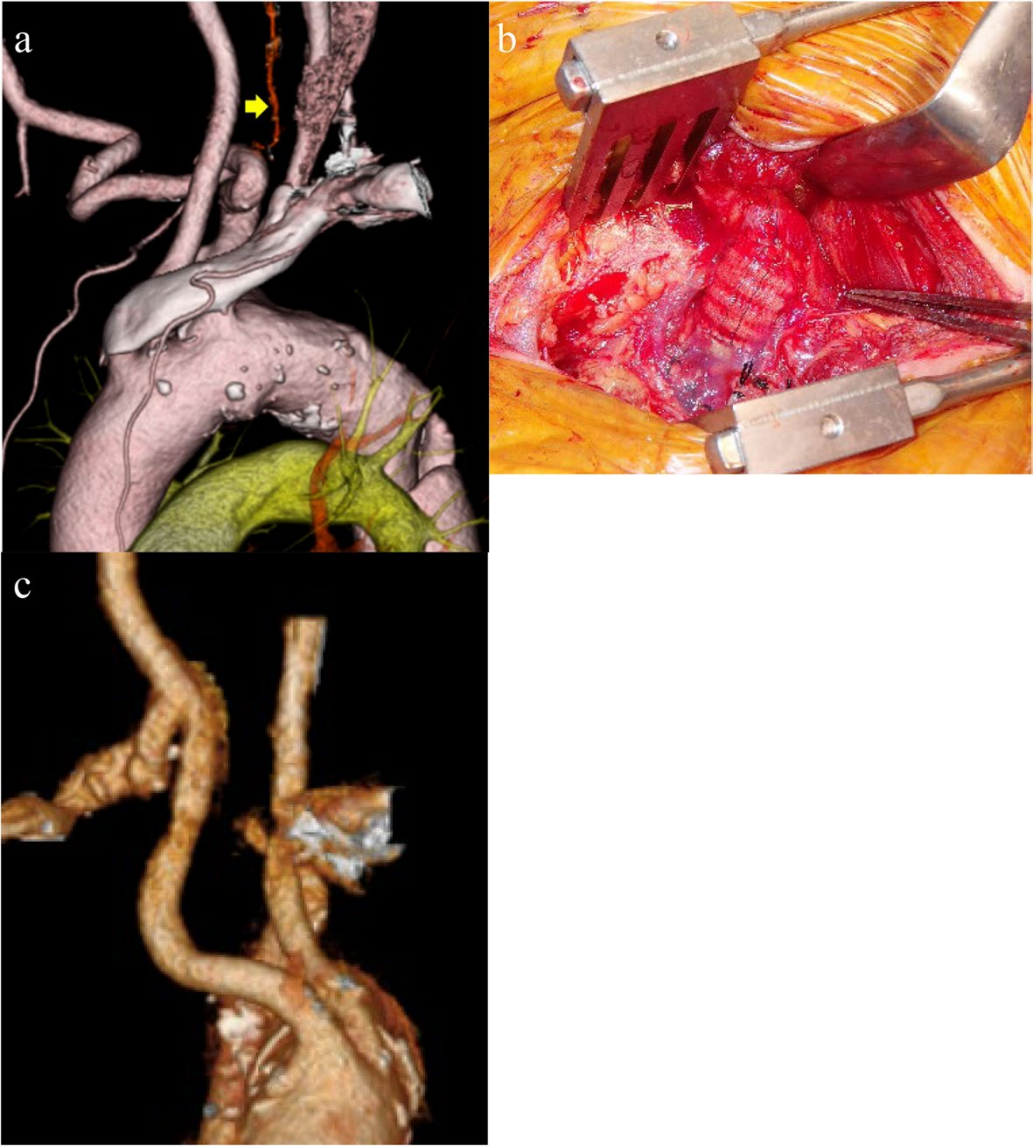
**a** 3D computed tomography reconstruction shows the right vertebral artery branched from the aberrant right subclavian artery (yellow arrow). **b** Intraoperative findings: right carotid artery-to-aberrant right subclavian artery bypass using an 8-mm Dacron graft (black arrow). **c** Postoperative computed tomography reveals a patent bypass graft (White arrow)

Six weeks after the abdominal aortic surgery, open surgery for the thoracic aortic lesion was scheduled with a two-stage approach. During the first operation, an RCCA-to-ARSA bypass was established using an 8-mm vascular graft (Triplex 8 mm TERUMO Corporation, Tokyo, Japan) through a right supraclavicular incision, and the ARSA was ligated proximal to the origin of the right vertebral artery (Fig. 2). Two days later, replacement of the thoracic aorta was performed as the second operation. The patient’s upper body was placed in the right lateral position and the lower body in a 45° semilateral position. A left anterolateral thoracotomy was performed in the fourth intercostal space, and an upper median sternotomy was conducted. Cardiopulmonary bypass was established by cannulation to the right femoral artery, left axillary artery, and right common femoral vein. The left ventricle was vented through the right upper pulmonary vein, and myocardial protection was achieved with cold blood cardioplegia. Under deep hypothermic circulatory arrest (bladder temperature, 25°C), the distal arch and descending aorta were opened after clamping the LSA, and selective cerebral perfusion was initiated through the left and right common carotid arteries and LSA. From within the aorta, the primary intimal tear of the dissection was detected on the proximal descending aorta between the orifice of the LSA and that of the ARSA. The orifice of the ARSA was located 2 cm distal to that of the LSA, but no luminal dilatation of the ARSA suggestive of a KD was found. Thus, the enlarged origin of the ARSA seen on preoperative CT was considered to be caused by an extension of the aortic dissection, not by a KD. The thoracic aorta was replaced using a 28-mm vascular graft with a 10-mm sidearm (Jgraft 28 mm; Lifeline Corporation, Tokyo, Japan), and the enlarged origin of the ARSA was partially resected to eliminate esophageal compression. The remaining aneurysmal wall was closed with a continuous suture. The distal aortic anastomosis was performed by a double-barreled procedure below the descending aortic aneurysm. Proximal anastomosis was performed between the left carotid artery and LSA. After de-clamping the ascending aorta, the LSA was reconstructed during rewarming.

**Fig. 3:**
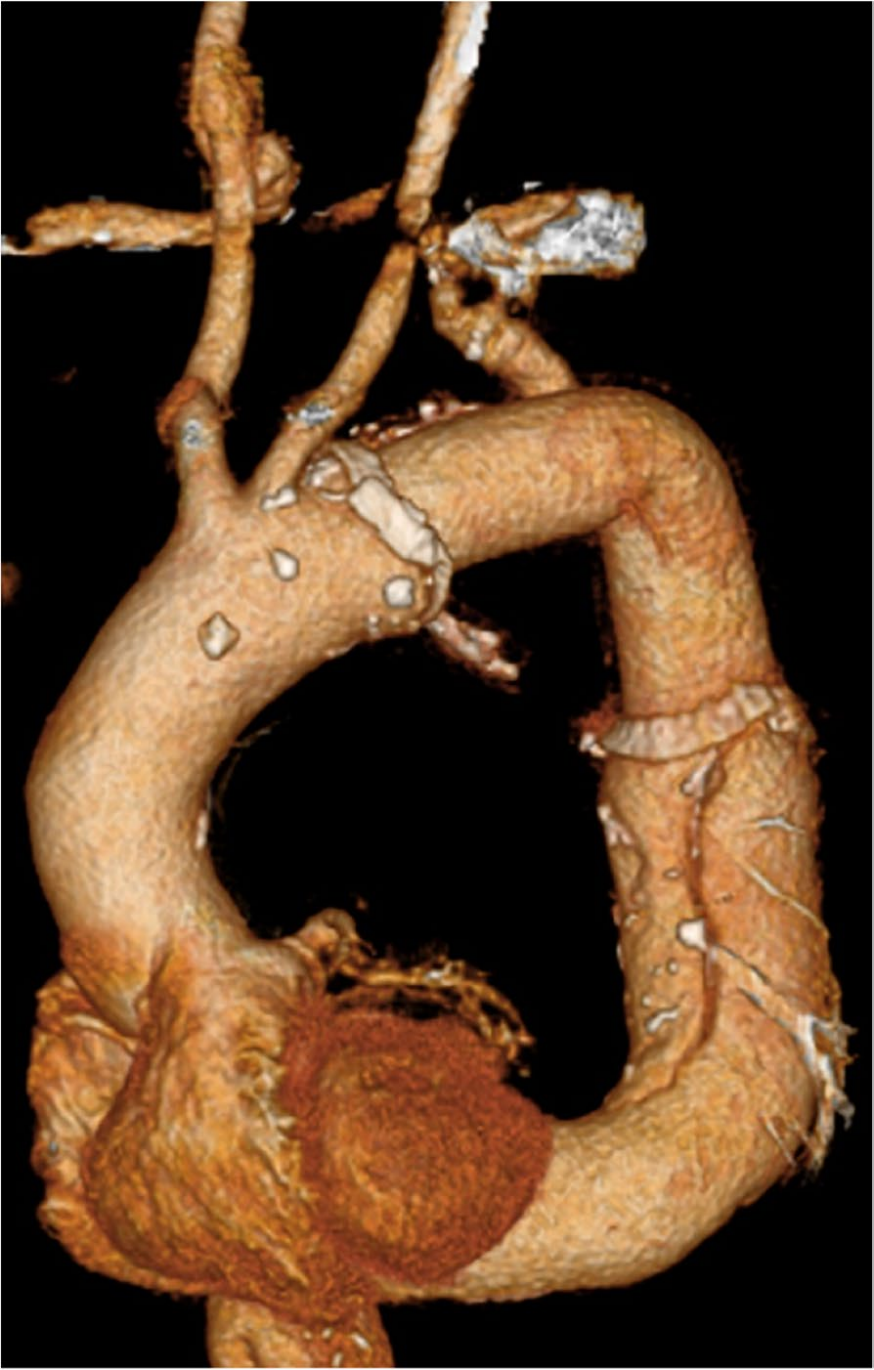
Postoperative computed tomography image after aberrant subclavian artery reconstruction and graft replacement

The patient was extubated 3 days postoperatively. His postoperative recovery was uneventful without any complications. Postoperative CT revealed favorable blood flow to the right and left subclavian arteries and successful replacement of the thoracic descending aorta without anastomotic leakage (Fig. 3). The patient was discharged on postoperative day 20. During a 3-year follow-up, hemodialysis was introduced due to worsening of preexisting renal dysfunction, but no clinical events or abnormal CT manifestations of the aorta were found. Histopathological examination of specimens of the descending aorta revealed atherosclerotic changes in the vascular walls.

## Discussion and conclusions

Embryologically, an ARSA results from regression of the right fourth aortic arch between the carotid and subclavian artery, such that the right seventh intersegmental artery and right dorsal aorta caudal to it continue as the right subclavian artery [4]. Up to 60% of ARSAs are associated with an enlarged origin arising from the aortic arch, called a KD. The KD is considered a remnant of a residual right dorsal aorta [2]. Approximately, 10% of patients with an aberrant subclavian artery have associated aortic pathology, such as aortic aneurysms or dissections [2]. Although the accurate pathogenesis of the associated aortic pathology is unclear, the acute angle take-off of an ARSA origin [5] and histological abnormalities of the remnant right dorsal aorta [3] have been speculated to increase the risk of dissection and aneurysm.

Type B dissection co-existing with ARSA is relatively uncommon. A total of 85 patients with type B dissection combined with ARSA have been described in the literature until February 2021 [1]. To manage this complex lesion, various surgical techniques, such as thoracic endovascular aortic repair, hybrid approach, and open surgery, including the frozen elephant trunk technique, have been employed [1]. With rapid advancements in endovascular technology, total endovascular or hybrid procedures are increasingly applied. However, there is currently no standard surgical approach due to the rarity of this lesion. According to a previous review [1], no significant differences were identified in in-hospital mortality or major or minor complications between these three approaches, and all three approaches demonstrated satisfactory short- and mid-term results.

In the current case, we employed open surgery using a two-stage approach [6] to eliminate esophageal compression from the enlarged and dissected origin of the ARSA and replace the coexisting descending aortic aneurysm and dissection. This approach was utilized due to reported cases of new-onset dysphagia secondary to a dissecting ARSA associated with type B acute dissection and aorto-esophageal fistula formation after exclusion of a KD aneurysm with an endograft [7, 8].

As the first operation, RCCA-to-ARSA bypass and ligation of the ARSA were performed. Although ligation of the subclavian artery is generally well tolerated in children, complications, including upper extremity ischemia, subclavian artery steal syndrome, and stroke, have been reported. A report of 14 adult patients [6] reported significant ischemic complications, such as coldness, rest pain, and fingertip necrosis, in 64% of those treated without restoration of blood flow to the upper extremity. In our patient, the bypass grafting and ligation of the ARSA allowed for restoration of blood flow to the upper extremity and prevention of peripheral embolism and stroke resulting from manipulation of the enlarged ARSA with thrombosis in the false lumen during the second operation. Additionally, ligation of the ARSA helped maintain a bloodless operative field during resection of the enlarged origin of the ARSA, and aortic reconstruction as blood backflow from the ARSA was completely blocked [9].

We performed a replacement of the thoracic aorta 2 days after the RCCA-to-ARSA bypass. This 2-day interval was considered important to avoid the complications and potential risk of bilateral recurrent laryngeal and phrenic nerve injuries, which may cause catastrophic respiratory compromise after repair of the thoracic aortic lesion with cardiopulmonary bypass. As the second operation, the thoracic descending aorta was approached through the left thoracotomy incision. This approach provided satisfactory exposure of the proximal portion of the ARSA, LSA, distal aortic arch, and descending thoracic aorta. It allowed resection of the enlarged ARSA and replacement of the distal aortic arch and proximal descending aorta without difficulty. The operative results of open surgery in the presence of an ARSA using cardiopulmonary bypass have been reported with mortality rates of 9–18%; however, recent reports have shown that with the establishment of conventional and frozen elephant trunk techniques in patients with ARSA pathologies, the mortality has decreased to 4.5% [1].

In conclusion, we report a case of coexistent type B aortic dissection and ARSA in a 72-year-old man. This complex lesion can be successfully repaired by open surgery with a two-stage approach, RCCA-to-ARSA bypass followed by thoracic aortic replacement.

## Data Availability

The datasets supporting the conclusions of this article are included within the article.

## Abbreviations

ARSA: Aberrant right subclavian artery
RCCA: Right common carotid artery
KD: Kommerell diverticulum
CT: Computed tomography
LSA: Left subclavian artery
